# Effect of drying, blanching, pickling and maceration on the fate of ^40^K, total K and ^137^Cs in bolete mushrooms and dietary intake

**DOI:** 10.1007/s11356-021-15523-9

**Published:** 2021-08-02

**Authors:** Jerzy Falandysz, Daniela Meloni, Alwyn R. Fernandes, Michał Saniewski

**Affiliations:** 1 Department of Toxicology, Faculty of Pharmacy, Medical University of Lodz, 1 Muszyńskiego Street, 90-151, Lódź, Poland; 2 Istituto Zooprofilattico Sperimentale del Piemonte Liguria e Valle d’Aosta, Via Bologna 148, 10154 Torino, Italy; 3 School of Environmental Sciences, University of East Anglia, Norwich, NR4 7TJ UK; 4 Institute of Meteorology and Water Management – National Research Institute, 42 Waszyngtona Av, 81-342 Gdynia, Poland

**Keywords:** Food analysis, Food composition, Edible fungi, Mushrooms, Foraged food radioactive contamination, Dietary exposure

## Abstract

The effects of blanching, blanching and pickling and maceration on the leaching of ^137^Cs and ^40^K from the flesh of three edible bolete mushroom species—*Boletus edulis*, *Leccinum scabrum* and *Leccinum versipelle*—were investigated. Significant (*p* < 0.05) decreases in activity were observed but varied depending on the treatment. Relative to fresh mushrooms, blanching decreased the activity concentration of ^137^Cs by 15 ± 13%, and of ^40^K, by 16 ± 7%, but blanching and pickling (vinegar) reduced activity more effectively, by 55 ± 8% and 40 ± 20% respectively. The corresponding losses of ^137^Cs and ^40^K through maceration of dried, powdered mushrooms were 38 ± 11% and 35 ± 14% ww, respectively. These results indicate that traditional domestic processing methods may not be as efficient at excluding ^137^Cs radioactivity as shown in some other studies. The activity concentration of ^137^Cs in a typically sized (100 g) portion of a processed mushroom (sourced from nearshore regions of the southern Baltic Sea coast near Gdańsk in 2015) meal was projected to be low, i.e. in the range of 0.51 to 12 Bq kg^−1^ ww. The corresponding effective dose of ^137^Cs from blanched, blanched and pickled and macerated mushrooms per capita was also assessed to be low, from 0.001 to 0.010 μSv. Nutritionally, the median concentration of potassium (330 mg) in 100 g portions of blanched or pickled mushrooms would account for around 7% of the adequate adult daily intake.

**Graphical abstract:**

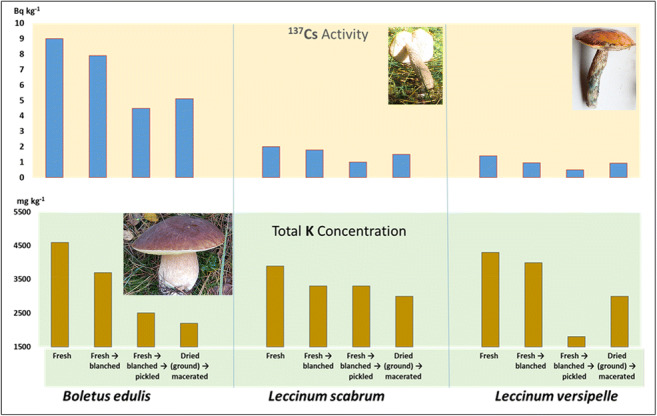

## Introduction

Edible wild mushrooms are efficient bio-accumulators of various mineral constituents and other beneficial organic compounds (e.g. antioxidants), which are vital for their enzyme-catalysed biosynthetic processes (Bhatia et al. 2013; Borovička et al. 2010; Falandysz 2013; Mędyk et al. 2020; Santiago et al. 2016). They are considered as nutritionally beneficial foods which in addition to improving bowel function, can also be a dietary source of essential macro-elements such as potassium (K), phosphorous (P) and micro-elements such as selenium (Se), zinc (Zn) and copper (Cu) (Falandysz and Borovička 2013; Nnorom et al. 2019). Some edible mushrooms from the genus *Boletus* are rich in Se and caps of the parasol mushroom *Macrolepiota procera* (Scop) Singer are rich in Cu (Falandysz 2013; Gucia et al. 2012; Stefanović et al. 2016). A number of species can also accumulate toxic elements such as arsenic (As), cadmium (Cd), mercury (Hg) and lead (Pb) in their edible fruiting bodies (Doğan et al. 2006; Zhang et al. 2020).

There is a long tradition in the cuisine and health practices of Asian, European and Latin American cultures of using preserved (by drying, souring and pickling) mushrooms (Gargano 2019; Lowenhaupt Tsing 2015; Santiago et al. 2016). The factors limiting the use of fresh wild mushrooms are the immediate availability of the fresh product, tradition or price. Mushrooms, fresh or preserved, need to be well cooked and meals prepared from wild species are exceptionally prized for their taste (Laessoe et al. 1996). There are also some very rare examples from traditional gourmet cultures around the world, where mushrooms are eaten raw, such as the matsutake mushroom, *Tricholoma matsutake* (S. Ito & S. Imai) Singer, i.e. consumed raw, either shredded or sliced, in Japan or China. The widely cultivated button mushroom, *Agaricus bisporus* (J.E. Lange) Imbach, can also be found uncooked in some salads.

Another unfortunate and long-lived contaminant that arises from anthropogenic activity is radiocaesium (^137^Cs), which also bio-accumulates in mushrooms in certain areas. As an environmental contaminant, radiocaesium occurs as two isotopes, ^137^Cs and ^134^Cs, but the latter is relatively short-lived (half-life of 2.06 years) and is generally used as a tracer of fresh emissions. As it is rarely detected in foraged or cultivated mushrooms, except immediately after contamination incidents, this study will mainly address ^137^Cs. The major source of radiocaesium contamination is radioactive fallout, originally from nuclear weapons testing and nuclear devices during 1945–1980 and later, from the Chernobyl accident in the Ukraine in 1986. Twenty-five years later, a nuclear accident in the Fukushima Daiichi nuclear power plant, in Japan, provided a more recent source. The accident released high levels of radioactivity, including radiocaesium which was later detected in the local wild mushrooms (Orita et al. 2017b; Prand-Stritzko and Steinhauser 2018). Following these incidents, a significant amount of research activity (Bem et al. 1990; Betti et al. 2017; Cocchi et al. 2017; Falandysz et al. 2015, 2018, 2019b; Grodzynska 2018; Grueter 1971; Klán et al. 1988; Mietelski et al. 1994; Orita et al. 2017a; Rantavara 1987; Steinhauser et al. 2014; Stijve and Poretti 1990; Strumińska-Parulska et al. 2021; Strumińska-Parulska and Falandysz 2020; Yoshida and Muramatsu 1994) was directed towards the radioactive contamination of foods, including wild mushrooms.

Culinary processing, as well as commercial processing of mushrooms can affect the concentration of mineral constituents to different extents, depending on the recipes or the processing technologies used (baking, blanching, boiling—hotpot, braising, deep oil stir-frying, deep freezing, frying, grilling, parboiling, pickling, roasting, stewing, autoclaving, etc.). These processes can be species dependent and are known to modify the nutritional values of mushroom meals (Barnett et al. 1999; Consiglio et al. 1990; Falandysz et al. 2019a; Nabeshi et al. 2013) and also subsequently affect the dietary intake of inorganic contaminants and the associated benefits or health risks. There are different ways of preparing wild mushrooms for consumption depending on the foraged species, which results in differences in texture and including firmness, elasticity, cohesion or wateriness between the prepared flesh of individual species. These differences coupled with the variety of kitchen recipes and the local gourmet culture can result in a range of different mineral intakes and exposure scenarios. Other dominant factors that strongly influence these scenarios are regional differences in radioactive contamination, soil geochemistry and accessibility to wild mushrooms. Practically, significant differences in reported data for mushrooms and mushroom meals can also arise from the manner in which contaminant concentrations, intake and exposure are reported, e.g. on a wet “whole” weight (ww) or dry weight (dw) basis, because cooking/processing will generally result in a reduction (decrease) of concentration or intake (Daillant et al. 2013; Falandysz et al. 2020b; Skibniewska and Smoczyński 1999).

Gamma spectroscopy with a high purity germanium detector is a non-destructive quantitative technique that is widely used for the activity concentration measurements of natural and artificial radionuclides, e.g. ^40^K, ^134^Cs and ^137^Cs in materials, and for the extrapolation of total K from the level of measured ^40^K. The technique was used in this study to investigate the effects of common household procedures used to prepare meals from fresh or dried mushrooms.

The study aimed to assess the effect of blanching, blanching and pickling, and maceration on the activity concentrations of natural ^40^K (including total K) and artificial ^137^Cs in traditionally prepared foods and the potential dietary intake, by sequentially subjecting individual batches of mushrooms to these cooking and preservation procedures. In comparison to earlier reported work, this study considers realistic mushroom preparation procedures based on the common processes that foragers and other consumers of these mushrooms, actually use. This naturally leads to more realistic intake assessments. Given the scarcity of data on activity levels in edible mushrooms, the use of samples collected in the wild in this study also provides an update on the current status of contamination in this popular food species, as well as exposure estimates for these consumer populations. Three species of mushrooms were selected for the study: the king bolete mushroom *Boletus edulis* Bull., and two species from the genus *Leccinum*: *L. scabrum* (Bull.) Gray and *L*. *versipelle* (Fr.& Hök) Snell)—all collected from the same region and during the same period. The intakes of ^137^Cs and total K were also estimated using hypothetical meals made from *B*. *edulis* and *Leccinum* spp. (blanched and blanched and pickled) mushrooms.

## Materials and methods

### Sample collection

The following samples were collected from a forested area (nearshore landscaped parkland) off the coastal Baltic Sea region of Pomerania Voivodeship (Pomerania province, Poland) in September 2015. *B*. *edulis* (8 well developed specimens—large with a white to yellow hymenophore), *L*. *scabrum* (16 well developed specimens—relatively large with white hymenophores) and *L*. *versipelle*, fruiting bodies (8 well developed specimens—large with white hymenophore). On collection, fresh fruiting bodies were immediately (on site) cleaned from any foreign debris (the lower part of stipe was cut-off uniformly) and stored in a wicker basket. All fruiting bodies within a species were collected in the same morning. At the laboratory, all freshly collected mushrooms were weighed and processed within 3–4 h of collection. Using a ceramic knife, each fruiting body was separated into cap and stipe which were further divided into two halves of a similar size, pooled and weighed separately (2 pools of halved caps and 2 pools of halved stipes) to provide four sets per species. One set of caps and one of stipes, per species, were deep frozen and then freeze-dried and further used as reference (control) material as well as for the maceration experiment. The remaining sets of pooled fresh halves of caps and stipes were used for the experiments investigating the effects of blanching and pickling.

### Freeze drying

For each species, the sub-sampled sets of caps and stipes were deep frozen separately at – 20 °C for 48 h, then freeze-dried (lyophilizer model LYOVAC GT2; Steris, Germany), weighed, packed in clean, unused sealed polyethylene bags and further ground to a fine powder using a porcelain mortar. The dehydrated and powdered samples of each fungal material were divided into two portions which served both as a reference (control) material for analyses and as a substrate for experiments aimed at determining the impact of maceration.

### Blanching and pickling

The procedures used for blanching (tap water without salt) and pickling of the fresh fungal materials with acetic marinade (the marinade was made by 1:4, mL:mL, dilution of spirit vinegar of 10% acidity with tap water in glass beakers) were similar to those presented by Drewnowska et al. (2017a, b). In brief, each material was gently boiled for 15 min using 150 mL tap water in a 250 mL beaker, with a 1:5 ratio of material to water. After draining off the liquid, the blanched materials were weighed and divided into two parts. One was deep frozen (− 20 °C), lyophilised, ground and stored in a screw capped plastic tube (capacity 15 mL, VWR®, Ultra High Performance; VWR, Radnor, Pennsylvania, USA) for instrumental analyses. The second part of the blanched material was used for the next stage of pickling. The materials were pickled using a solution of spirit vinegar marinade in 150 mL beakers, covered with laboratory foil and maintained at room temperature (19 °C) for 30 days. At the end of this period, the pickled materials were drained, frozen, lyophilised, ground and stored in clean screw capped plastic tubes until gamma spectroscopy analysis.

### Maceration (soaking)

Sub-portions of the pooled and powdered caps or stipes (ca 1 g each per species) were cold macerated for 24 h at room temperature in 100 mL glass beakers using 50 mL of deionized water (Drewnowska et al. 2017a). The macerate was separated from fungal solids by filtration under gravity, through a medium fine laboratory filter paper in a plastic funnel. The filtered fungal solids were pre-dried at room temperature for 24 h followed by a further 24 h at 65 °C in a laboratory oven. The dried materials were powdered in a porcelain mortar, transferred into screw capped plastic tubes, closed and stored in clean and dry conditions for further analyses.

### Determination of ^137^Cs and ^40^K activity concentrations by gamma spectrometry

Immediately prior to instrumental analysis, the fungal materials that were not already prepared for analysis were weighed, deep frozen and freeze-dried for three days (Labconco Freeze Dry System, Kansas City, MO, USA), then reweighed and homogenised so that the activity concentrations of ^137^Cs and ^40^K were determined in fully dehydrated materials.

The activities were determined using a gamma spectrometer with a coaxial HPGe detector and with a relative efficiency of 18% (Detector GC 1819 7500 SL, Canberra Packard, Poland, Warsaw). The resolution efficiency was 1.9 keV at 1.332 MeV (with associated electronics). The measurements of the fungal materials in this study were preceded by background measurements (time 80,000 s) using a similar counting time (> 22 h). The equipment was calibrated with a multi-isotope standard using validated methodology. The standard reference solution, “Standard solution of gamma emitting isotopes”, code BW/Z-63/48/16), obtained from the IBJ-Świerk near Otwock in Poland, was used for preparing solutions for equipment calibration. The radionuclides used in the reference solution during equipment calibration were ^241^Am (1.2%), ^109^Cd (2.1%), ^57^Co (0.80%), ^51^Cr (1.55%), ^113^Sn (2.0%), ^85^Sr (1.2%), ^137^Cs (1.5%), ^54^Mn (1.55%), ^65^Zn (1.2%) and ^60^Co (0.8%). The same geometry of cylindrical dishes with 40 mm diameter was used for the analysis of the fungal material extracts as well as for the reference samples during equipment calibration organised by IAEA-RML-2018-01. Detailed results of a successful inter-calibration study carried out during the course of this work have been reported earlier (Falandysz et al. 2020a, 2021b; Saniewski et al. 2020).

Minimum detectable activity (MDA) was determined by the Curie method. This method is based on two basic parameters: (i) critical level, which is defined as a level below which the detection signal cannot be reliably recognized and (ii) detection limit specifying the smallest signal that can be quantitatively reliable. The ^134^Cs activity concentrations were above MDA (3.64–4.85 Bq kg^−1^ dw) in most of the samples and for a few samples with mass below 2 g was 14 Bq kg^−1^ dw.

The means of ^137^Cs and ^40^K activity concentrations and of total K concentration for the whole fruiting bodies (including culinary processed products) were calculated both on a wet and dry weight basis, taking into account the biomass share of the caps and stipes in the whole fruiting bodies, fresh and dehydrated, per species, respectively. The concentration of stable K was calculated from the ^40^K data as used in other studies (Falandysz et al. 2020a; Samat et al. 1997). Dehydrated subsamples of freeze-dried caps and stipes of each mushroom species were used as control (reference) materials to calculate the change in the activity concentrations of ^137^Cs and ^40^K before and after culinary processing. Data obtained on activity concentration of ^137^Cs were decay corrected back to the time of sampling (Falandysz et al. 2020a). A free software (Social Science Statistics; www.socscistatistics.com) was used for statistical analyses.

## Results and discussion

**Fig. 1:**
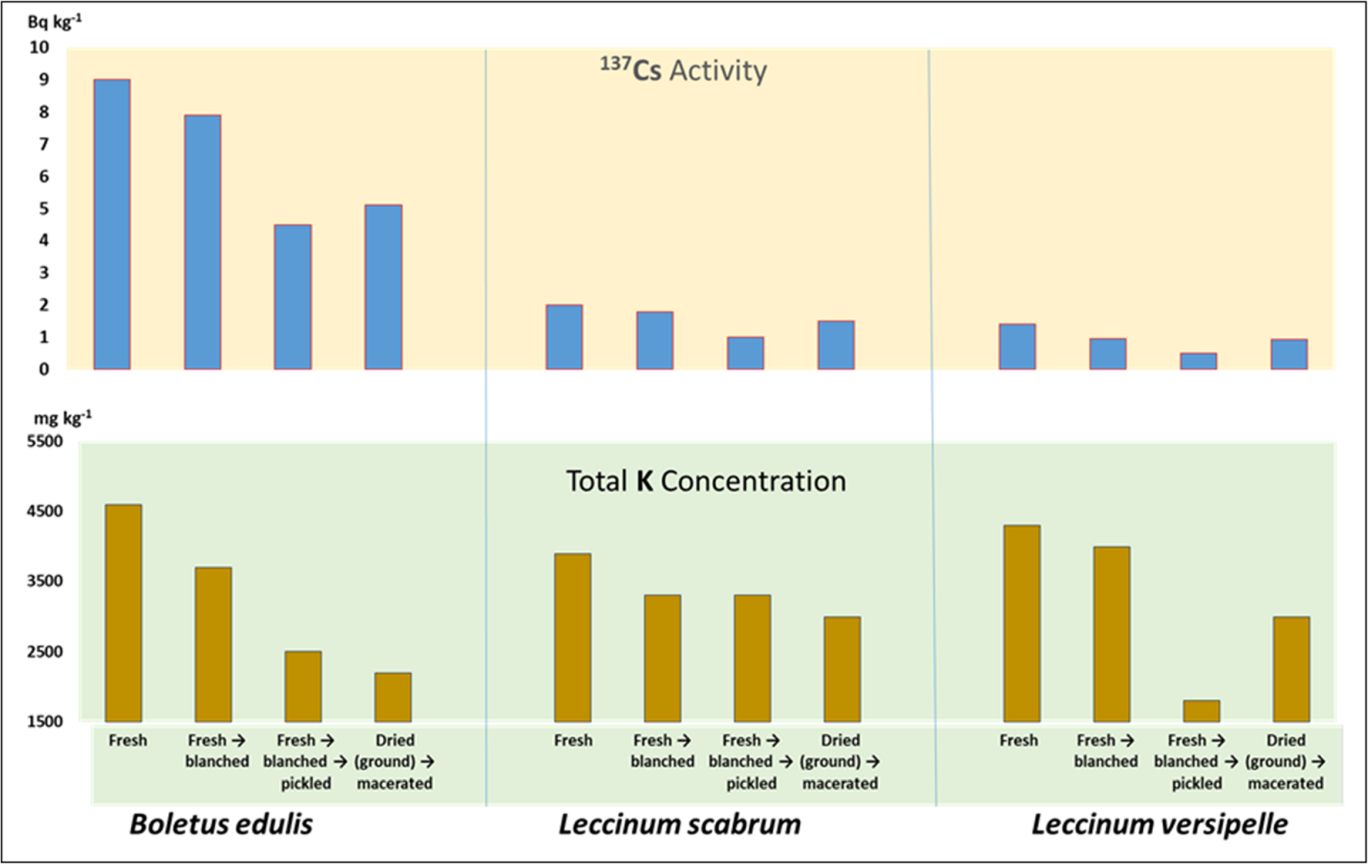
A graphical representation of effects on ^137^Cs activity concentration and total K concentration (on wet weight basis) in three edible bolete mushrooms after culinary processing

The results were expressed in Tables 1, 2, 3, 4, on a wet weight basis (to enable the estimation of the probable dietary intake through the consumption of mushroom meals), as well as on a dry weight basis (Appendix Tables 5, 6, 7), to allow comparison with some literature data where wet weight concentrations are not provided (Beresford et al. 2001; Consiglio et al. 1990; Daillant et al. 2013; Kenigsberg et al. 1996; Nabeshi et al. 2013; Rantavara 1987; Shutov et al. 1996; Skibniewska and Smoczyński 1999; Steinhauser and Steinhauser 2016; Stijve 1994). A graphical representation of the effects on ^137^Cs activities and total K concentrations (ww) in edible wild mushrooms after culinary processing has been presented in Fig. 1. In all cases, the process used has resulted in reduction, with the greatest losses seen for the combination of blanching and pickling (^137^Cs) and maceration (for K in two of the three species).

**Table 1:** ^40^K and ^137^Cs activity concentration in fresh (lyophilised) and culinary processed caps, stipes and whole fruiting bodies of bolete mushrooms (Bq kg^−1^ on wet weight basis)

Species and kind of a culinary process	^40^K (Bq kg^-1^ ww)			^40^K; effect (decrease in %)			^137^Cs (Bq kg^−1^ ww)			^137^Cs; effect (decrease in %)		
	Caps	Stipes	Whole mushrooms	Caps	Stipes	Whole mushrooms	Caps	Stipes	Whole mushrooms	Caps	Stipes	Whole mushrooms
*Boletus edulis* (8)^#^												
Fresh → lyophilised ^*^	130 ± 12	130 ± 17	130 ± 14	NA	NA	NA	11 ± 1	8.3 ± 0.4	9.0 ± 1.0	NA	NA	NA
Fresh → blanched	128 ± 14	72 ± 35	110 ± 22	1.5	45	23	8.4 ± 0.4	7.0 ± 0.5	7.9 ± 0.4	24	16	12
Fresh → blanched → pickled	84 ± 6	50 ± 18	70 ± 12	35	45	46	4.8 ± 1.0	3.9 ± 0.3	4.5 ± 0.7	56	53	50
Fresh → lyophilised (ground) → macerated	66 ± 10	59 ± 21	63 ± 15	49	55	51	6.9 ± 0.3	2.5 ± 0.3	5.1 ± 0.2	37	70	43
*Leccinum scabrum* (16)^#^												
Fresh → lyophilised ^*^	120 ± 11	100 ± 13	110 ± 12	NA	NA	NA	2.6 ± 0.2	1.0 ± 0.2	2.0 ± 0.2	NA	NA	NA
Fresh → blanched	90 ± 16	97 ± 24	92 ± 19	25	3.0	16	2.5 ± 0.3	< 1.8	~ 1.8	3.8	WD	~ 3.8
Fresh → blanched → pickled	87 ± 23	92 ± 48	90 ± 33	27	8.0	18	1.4 ± 0.3	0.55 ± 0.32	1.0 ± 0.3	46	45	50
Fresh → lyophilised (ground) → macerated	110 ± 25	44 ± 13	84 ± 20	8.3	56	24	2.0 ± 0.4	0.77 ± 0.16	1.5 ± 0.3	23	23	25
*Leccinum versipelle* (8)^#^												
Fresh → lyophilised^*^	120 ± 12	110 ± 14	120 ± 13	NA	NA	NA	1.7 ± 0.2	1.0 ± 0.2	1.4 ± 0.2	NA	NA	NA
Fresh → blanched	100 ± 28	120 ± 18	110 ± 24	17	+ 9.1	8.3	1.2 ± 0.3	< 1.1	~ 0.95	29	WD	~ 29
Fresh → blanched → pickled	45 ± 25	58 ± 17	51 ± 22	62	47	57	1.5 ± 0.4	0.54 ± 0.41	0.51± 0.40	12	46	64
Fresh → lyophilised (ground) → macerated	100 ± 10	62 ± 12	85 ± 11	17	44	29	0.98 ± 0.18	0.88 ± 0.18	0.94 ± 0.18	48	33	45

Notes: ^#^(composite samples with amount of fruiting bodies given in parentheses); ^*^Reference material; NA (not applicable); WD (without data)

**Table 2:** Potassium concentration in fresh (lyophilised) and culinary processed caps, stipes and whole fruiting bodies of bolete mushrooms (mg kg^−1^ wet weight basis)

Species and kind of a culinary process	K		
	Caps	Stipes	Whole mushrooms
*Boletus edulis*			
Fresh → lyophilised	4,600	4,600	4,600
Fresh → blanched	4,500	2,600	3,700
Fresh → blanched → pickled	3,000	2,500	2,500
Fresh → lyophilised (ground) → macerated	2,400	2,100	2,200
*Leccinum scabrum*			
Fresh → lyophilised	4,300	3,600	3,900
Fresh → blanched	3,200	3,400	3,300
Fresh → blanched → pickled	3,000	3,300	3,300
Fresh → lyophilised (ground) → macerated	3,900	1,600	3,000
*Leccinum versipelle*			
Fresh → lyophilised	4,300	3,800	4,300
Fresh → blanched	3,600	4,400	4,000
Fresh → blanched → pickled	1,700	2,100	1,800
Fresh → lyophilised (ground) → macerated	3,600	2,200	3,000

**Table 3:** Integrated data on the effect of culinary processing on activity concentrations of ^40^K and ^137^Cs in *B*. *edulis*, *L*. *scabrum*, and *L*. *versipelle* (percentage of decrease; mean ± S.D.; wet weight basis)

Kind of a culinary process	Effect decrease in %					
	^40^K			^137^Cs		
	Caps	Stems	Whole mushrooms	Caps	Stems	Whole mushrooms
Fresh → blanched	14 ± 12	13 ± 27	16 ± 7	19 ± 13	16	15 ± 13
Fresh → blanched → pickled	41± 18	33 ± 22	40 ± 20	38 ± 23	48 ± 4	55 ± 8
Fresh → lyophilised (ground) → macerated	25 ± 21	52 ± 7	35 ± 14	36 ± 12	42 ± 25	38 ± 11

**Table 4:** Estimated annual internal radiation dose (μSv) per capita from decay of ^137^Cs from 100 g (wet weight basis) portions of mushroom products

Species and type of treatment	Internal radiation dose (μSv)
*Boletus edulis* (whole fruiting bodies)	
Fresh	0.012
Fresh → blanched	0.010
Fresh → blanched → pickled	0.006
Dried (ground) → macerated	0.007
*Leccinum scabrum* (whole fruiting bodies)	
Fresh	0.003
Fresh → blanched	0.002
Fresh → blanched → pickled	0.001
Dried (ground) → macerated	0.002
*Leccinum versipell*e (whole fruiting bodies)	
Fresh	0.002
Fresh → blanched	0.001
Fresh → blanched → pickled	0.001
Dried (ground) → macerated	0.001

### ^40^K, total K and ^137^Cs in uncooked mushrooms

In this study, the mushrooms showed relatively low activity concentrations of ^137^Cs compared to those of ^40^K in caps, stipes and whole fruiting bodies (Tables 1 and 2). The activity concentration of ^40^K in whole fruiting bodies of *B*. *edulis*, *L*. *scabrum* and *L*. *versipelle* were in the range from 110 to 130 Bq kg^−1^ ww (Table 1). Drying of mushrooms (usually sliced) in typical domestic preparation or preservation conditions, i.e. air drying at ambient temperature and in sunshine or in an oven or electrically heated commercial dryer at 40 °C to 60 °C, removes most of the moisture and some volatiles, leaving behind the dried solid flesh.

Parameters such as hardness, cohesion and wateriness that are typical for mushroom species can be the major factors determining the leaching rate of radionuclides and other mineral constituents during culinary processing of the fruiting bodies but this aspect has been little studied so far. The typical moisture (water, humidity) content of fresh fruiting bodies of the firmer fleshed *B*. *edulis* that are most suitable for consumption is close to 90.0% (Falandysz et al. 2021a). Similar values have been reported in other studies (Jaworska and Bernaś 2009; Kenigsberg et al. 1996), and this is also the consensus value for wild edible mushrooms, in general. The reported moisture content of blanched *B*. *edulis* in different studies were 90.44% (Jaworska and Bernaś 2009); 78.20 ± 0.43% (Jaworska et al. 2015) and 86.2% (range 85.4 to 86.7%) for commercially pickled mushrooms (*B*. *edulis*, *Imleria badia* and *Suillus luteus*) (Saba and Falandysz 2021).

Recalculation of data for mineral constituents in dried mushrooms to a wet weight basis generally uses a factor of 10, by consensus, although some authors have reported using a factor of 10.5 (Kalač 2013; Kenigsberg et al. 1996). Contaminant concentrations in freeze-dried products in the present study were basically 10-fold higher due to the effect of dehydration (Appendix Table 5 and 6), which is the maximal value of increase.

Typical traditional recipes suggest that fruiting bodies should be rinsed with tap water and dried completely (with a towel and laid out to air-dry for a few minutes) before culinary processing, in order to obtain a texture that is “crisp, buttery and savoury, instead of getting rubbery and spongy”. This can impact on the results of determination of any mushroom contaminants (or nutrients) studied. Data reporting occurrence of minerals, trace elements and radionuclides in different species of raw mushrooms are usually expressed or normalised on a dry weight basis. However, the estimation of dietary intake and human exposure requires all components of the calculation (original weight, weight after culinary processing, weight of the consumed product as well as residual water or absorbed fat/oil in fried mushroom meals) to be wet or fresh weight, so in these cases, ww data are more appropriate.

The potassium concentration in the whole fruiting bodies of *B*. *edulis* was 4,600 mg kg^−1^ ww, and ranged from 3,900 to 4,300 mg kg^−1^ ww in the *Leccinum* spp. (Table 2). ^40^Potassium (thus also total K) was more evenly distributed between the caps and stipes than ^137^Cs, with mean quotient (Q_C/S_) values ranging between 1.0 and 1.2 (Appendix Table 5). The Q_C/S_ values for K in *B*. *edulis* in this study roughly agree with published data (Frankowska et al. 2010; Zhang et al. 2010), for the inland regions of Poland which show Q_C/S_ ratios of 1.5 to 1.6 (median values). The corresponding median values of Q_C/S_ for several sets of *L*. *scabrum* from the inland regions of Poland were in the range 1.4 to 2.7 (Falandysz et al. 2018, 2021c). A recent study showed that the distribution of K between the cap and stipe of fruiting bodies of *Amanita muscaria* (L.) Lam. varied depending on the stage of development of the mushroom. Mature fruiting bodies showed a greater abundance of K in the stipes when compared to immature “button stage” sized and juvenile specimens, with Q_C/S_ values of 0.62 to 1.2 and 1.4 to 1.6 respectively. Therefore, the values of Q_C/S_ for ^40^K obtained in this study for mature fruiting bodies (Appendix Table 5) agree with those for *A*. *muscaria* at a similar developmental stage. Potassium is the major metallic element in the flesh of mushrooms (Stijve 1996). Mushrooms in this study (Table 2, Appendix Table 6) showed similar total K concentrations as reported in other studies for *B*. *edulis* and *L*. *scabrum* (Falandysz et al. 2018; Zhang et al. 2010).

The activity concentration of ^137^Cs in whole *B*. *edulis* was 9.0 Bq kg^−1^ ww (90 Bq kg^−1^ dw). Individual King Bolete specimens in this study, which showed ^137^Cs cap concentrations of 110 kg^−1^ dw (Appendix Table 5), were, from the radio-toxicological point of view, much less contaminated with this nuclide than individuals collected in nearby areas (50–70 km southwest) in 2007 (1,400 ± 17 Bq kg^−1^ dw in caps), and in 2010 (500 ± 9 Bq kg^−1^ dw in caps) (Falandysz et al. 2015, 2021b). This agrees well with recent, long-term studies on the accumulation of ^137^Cs by mushrooms such as Gypsy *Cortinarius caperatus* (Pers.) Fr. and Common Chanterelle *Cantharellus cibarius* Fr., which have shown a slow long-term decline in activity concentration in a serrated pattern (Falandysz et al. 2016, 2019b) which arises from the smaller seasonal variability, time of sampling and possibly also from the biological and ecological impacts on contaminant uptake by the mycelium. The mycelial network of *B*. *edulis* penetrates deeper into the soil horizon and mushrooms collected in Poland in the period 1986–2019 have shown a higher contamination of fruiting bodies around 10 to 20 years after the 1986 Chernobyl incident, i.e. in the period 1995–2010 (Falandysz et al. 2021b).

The *Leccinum* mushrooms were less contaminated, with ^137^Cs, at levels of 2.0 Bq kg^−1^ ww (20 Bq kg^-1^ dw) in *L*. *scabrum*, down to 1.4 Bq kg^−1^ ww (14 Bq kg^−1^ dw) in *L*. *versipelle* (Table 1, Appendix Table 5). Literature observations (Cocchi et al. 2017; Falandysz et al. 2021b) show that typically, ^137^Cs occurs to a greater extent in the caps than stipes for boletus mushrooms, which agrees well with the observations made in this study, i.e. Q_C/S_ value of 1.3 for *B*. *edulis,* and 1.7–2.6 for *Leccinum* spp. (Appendix Table 5).

### ^40^K and ^137^Cs in mushrooms after blanching

For all the three studied species, ^40^K activities in blanched whole mushrooms decreased by 16 ± 7% ww (Table 3), or 38 ± 4 if expressed as dw (Appendix Table 7). The total K concentration in blanched whole mushrooms was in the range 3,300 to 4,000 mg kg^−1^ ww (Table 2). Fresh *B*. *edulis* when blanched lost ^40^K activity concentration by 8.5% ww (34% dw) and if deep frozen and then blanched, by 22% ww (44% dw) (Saba and Falandysz 2021).

The ^137^Cs activity concentrations in blanched whole boletes decreased by 15 ± 13% ww and by 39 ± 9% dw (Table 3, Appendix Table 7) with some variations for the species (Table 1, Appendix Table 5). These results confirm a finding by Daillant et al. (2013) who stated that “As regards the different experiments performed to try to extract parts of radiocaesium, most of them were disappointing compared with results available in literature”, i.e. that the removal of ^137^Cs using the typical cooking practice of blanching with fresh boiling water, is only partially successful, but has the advantage of retaining nutritionally important potassium. The study reported that *B*. *edulis* boiled (blanched) for 10 min lost little of ^137^Cs, and when frozen and then boiled, lost only 6.6% ww, while *Hydnum repandum* L. when boiled for 20 min lost 51% ww (Daillant et al. 2013).

The Chernobyl nuclear incident raised urgent questions about the short- and long-term safety of foods due to radiocaesium contamination and its effects in human following low levels of exposure (Venturi 2020). It had been anticipated that the consumption of wild mushrooms foraged in contaminated areas would contribute to human exposure to radioactivity, through dietary intake. Nutritionally however, wild mushrooms represent a significant component of the diet for some individuals (Barnett et al. 1999; Stijve 1994; Shutov et al. 1996; Zhang et al. 2010). Hence, it would be desirable to use cooking and preservation procedures that effectively decrease radiocaesium contamination while preserving the taste, texture, aroma and nutritional value.

Blanching of fresh mushrooms is a necessary step in some cooking recipes (with addition of the discarded water to soup, or during frying or pickling) but it can depend on the species and circumstances (type of meal, family or local customs). As an example, the Slippery Jack (*Suillus luteus*) always needs to be blanched before, e.g. flat pan frying, soup-making or pickling. Blanching (boiling) of mushrooms results in shrinkage of the fruiting bodies and loss of solutes (Biekman et al. 1996). Repeated and especially prolonged blanching can cause substantial depletion of water soluble nutrients, flavour, taste and texture (Dikeman et al. 2005), and probably also ^137^Cs.

During domestic preparation, blanching of wild mushrooms is typically carried out using boiling tap water (usually with the addition of a pinch of table salt) with the aim of removing excess glue polysaccharides (which will absorb water from the body if eaten and lead to dehydration), denaturing proteins/peptides and making the mushrooms more digestible. The addition of a chelating agent during blanching is not practiced domestically but may be used during commercial production of mushroom products.

Stijve et al. report on the efficiency of radiocaesium extraction from species such as *Cantharellus tubaeformis* (Fr.) Quél, *Hydnum repandum* L., *Hygrophorus camarophyllus* (Alb. & Schwein.) Dumée, Grandjean & Maire and *Albatrellus ovinus* (Schaeff.) Kotl. & Pouzar*,* through soaking or blanching with a water and salt solution (Stijve 1994). Extraction was more efficient from deep frozen and dried mushrooms (which causes partial disruption of the cell tissues), and also when a salt solution was used rather than using water alone (Stijve 1994). Similar results for deep frozen and dried mushrooms were noted in a study by Saba and Falandysz (2021).

Fresh *C*. *tubaeformis* mushrooms (large fruiting bodies were quartered while small ones were left whole) that were plunged into boiling water for 5 and 10 s lost 58 and 62% of radiocaesium respectively. Longer durations of 1, 5 and 10 min resulted in losses of 74, 83 and 89% respectively (Stijve 1994). Another batch blanched for 10 min (100 g in 0.5 L water) lost 51%, while under the same condition, other species such as *Albatrellus ovinus* lost 46%, *Hydnum repandum* lost 82% and *Hygrophorus camarophyllus* lost 88% (when boiled twice, it lost 97%) (Stijve 1994). Other metals such as Mn, Cu, Zn and Fe are also leached out during blanching and pickling (acid), as in the case of *A*. *bisporus*, where the rates of loss were 45, 3.9, 23 and 35%, respectively. The product also lost 37% of weight, but storage did not affect the elemental concentration or the weight (Coşkuner and Özdemir 1997).

Thus, the duration of blanching, the conditions used (water temperature, use of salt), any initial size reduction (chopping, slicing or left whole) and the species of mushroom used can substantially modulate the leaching rate of ^137^Cs. In the present study, the loss of potentially water-soluble monovalent Cs and K from blanched species was not too high and similar to results reported by Daillant et al. (2013), but generally at lower rates than those reported in other studies. On the other hand, elements occurring at a higher oxidation state than Cs and K, i.e. Mn, Cu, Zn and Fe and *A*. *bisporus*, were leached in a wider range.

### ^40^K and ^137^Cs in mushrooms blanched followed by pickling

Blanching followed by pickling of mushrooms results in hydrolysis, denaturation and partial disruption of cell walls and other structures as well as a chelating effect of vinegar. As expected in this study, the process resulted in increased leaching of both ^40^K (total K) and ^137^Cs from the mushrooms, as shown in Tables 1 and 3 (for dw data, also in Appendix Table 5 and 7). The losses of ^40^K (total K) and ^137^Cs after blanching and pickling were more pronounced than for blanching alone, rising for individual species from 18 to 67% ww (total 40 ± 20%) and from 50 to 64% ww (total 55 ± 8% ww), respectively (Tables 1 and 3). Our result is close to that for pickled (in the traditional way) *Xerocomus subtomentosus* (L.) Quél., which lost activity concentration of ^137^Cs by 58% ww (Skibniewska and Smoczyński 1999).

Dvořák et al. (2006) observed that fresh fruiting bodies of Bay bolete *Imleria badia* (Fr.) Vizzini (previous name *Xerocomus badius*), when immersed in a 2% solution of acetic acid (concentration relatively high for pickling) for 24 h, lost activity concentration of ^137^Cs by 61 to 58% ww and of ^40^K by 64 to 51% ww, while prolonged treatment increased the rate of loss until substantial change in the consistency of the flesh was observed. *A*. *bisporus* when blanched with the addition of ethylene diamine tetra-acetic acid preferentially lost some Fe and Cu but not Mn and Zn, but the addition of citric acid had no effect (Coşkuner and Özdemir 2000).

### ^40^K, total K and ^137^Cs, in mushrooms after maceration of fresh, dried and powdered fungal materials

Dried mushrooms (either whole, crushed or milled with partially destroyed cell walls) can be rehydrated (the absorption of water causes maceration which breaks down organised cell structures and loss of soluble solids) before further culinary processing, depending on the purpose and circumstances. Sometimes, dried or powdered mushrooms can be added directly to a cooked meal, e.g. in bigos (a traditional Polish hunter’s meat stew), and used in mushroom soups, crèmes and sauces, which results in maceration and includes both, the rehydrated mushrooms and the macerate.

Thus, rehydration of soaked dried mushrooms combined with the defragmentation resulting from fine milling in a kitchen mortar or a blender and depending on temperature can accelerate the leaching rate of organics and inorganics out of the substrate into the water phase. These processes can result in more effective exclusion of radiocaesium, provided of course that the macerate is rejected. Dried *B*. *edulis* when rehydrated absorbs ~ 55–65% of the fresh mushroom water, and retain ~ 45.4% of soluble solids of the initial dry weight (García-Pascual et al. 2005).

Following maceration, the activity concentrations of ^40^K and concentrations of total K in *B*. *edulis* and *Leccinum* spp. decreased relative to the fresh weight from 24 to 51% (Table 1), with an overall loss of 35 ± 14%, both in ww and dw (Table 3, Appendix Table 7). The activity concentration of ^137^Cs in macerated *B*. *edulis* mushrooms was 51 Bq kg^−1^ dw (5.1 Bq kg^−1^ ww, assuming full rehydration of the powdered product up to the original wet weight), with lower levels of 9.4 to 15 Bq kg^−1^ dw (0.94 to 1.5 Bq kg^−1^ ww) in *Leccinum* spp. This corresponded to an overall reduction of 38 ± 11% ww (33 ± 10% dw) of the ^137^Cs activity concentration (Table 3, Appendix Table 7).

A range of radiocaesium losses to the macerate, from soaked or rinsed, fresh and dried mushrooms, have been reported in the literature. As reported earlier (Stijve 1994), the proportion of fungal material that disintegrates during grinding is a major factor governing the leaching of minerals from cells. Another factor apart from temperature is the period of maceration, both for ground or dry sliced mushrooms, which traditionally can last from 2 h to overnight (8–12 h). In practice, rehydration is more efficient for sliced or powdered mushrooms, but less so for dried whole fruiting bodies which shrink by the greatest extent, reducing the ability of this product to rehydrate. However, experimental data (Stijve 1994; Vinichuk et al. 2005; Nabeshi et al. 2013), examining the leaching of ^137^Cs from macerated or soaked dried fungal materials (powdered or mostly crushed) still shows considerable variability.

In another example, fresh, quartered, fruiting bodies of *C*. *tubaeformis* soaked for 12 h (200 g in 3 L fresh water) lost 40% of radiocaesium, increasing to 50% and 61% respectively, when salted water (1 and 5% NaCl) was used, with little effect on the organoleptic qualities such as the taste, texture, colour and odour. The same material lost 95% of radiocaesium when rinsed two times, then blanched, but this process also resulted in a slimy consistency of the product (Stijve 1994).

Samples of dried, whole *C*. *tubaeformis* (16 g) when soaked for 30 min (0.5 L water), lost 40% of radiocaesium. Soaking for 15 min (0.5 L water) followed by blanching for 3 min resulted in a 99% loss of radiocaesium but the texture and taste of the reconstituted mushrooms were maintained (Stijve 1994). Soaking of dried Shitake mushroom *Lentinula edodes* (Berk.) Pegler., in water, decreased the radiocaesium activity concentration by around 50% in relation to uncooked shitake (Nabeshi et al. 2013).

In an experiment approximating to culinary maceration, ^137^Cs was extracted from dried (at 60–70 °C) and powdered fruiting bodies of eleven species of mycorrhizal mushrooms and two species of saprophytic mushrooms (Vinichuk et al. 2005). Then, 0.5 g portions of the fungal substrates were soaked in 30 mL of distilled water for 48 h at room temperature (one set of experiments), while a parallel experiment with the same substrates used hot (80 °C) water followed by agitation for 4 h (with half minute interruptions every 0.5 h) and subsequent filtration (Vinichuk et al. 2005). Water at room temperature extracted ^137^Cs at median (%) rates of 68 ± 11% (range 42–83%) from the mycorrhizal species, and 53 ± 19% (range 24–61%) from the saprotrophic species. The hot water was more efficient at extracting ^137^Cs at rates of 93 ± 6% (range 76–97%), and 70 ± 14% (range 58–86%), respectively. The study (Vinichuk et al. 2005) also showed species-dependent differences in the extraction efficiency of ^137^Cs and lower rates (using hot water) for *Tricholoma portentosum* (Fr.) Quél. (insoluble fraction at 13–24%), *Hypholoma sublateritium*–current name *Hypholoma lateritium* (Schaeff.) P. Kumm., (insoluble fraction at 29%) and *Armillariella mellea* (Vahl) P. Kumm. (insoluble fraction at 14–42%).

### Potential intake of ^137^Cs and total K from hypothetical mushroom meals

The potential intakes of ^137^Cs and total K through the consumption of mushroom meals were estimated, assuming that typically 100 g portions of cooked or processed mushrooms were consumed in a single meal. The ^137^Cs activity concentrations of these cooked mushrooms meals were projected to be very low, i.e. in the range of 0.094 ± 0.018 to 0.79 ± 0.04 Bq kg^−1^ ww. This range is considerably lower than the maximum permitted activity concentration of 600 Bq kg^−1^ for fresh mushrooms imported from third countries [the regulation applies to13 countries] to the European Union (EU 2020). The corresponding activity concentrations of ^40^K were substantially higher than those of ^137^Cs (Table 1).

Both raw mushrooms as well as mushroom meals contain relatively high amounts of potassium (Falandysz et al. 2020b; Stijve 1996), an element that undergoes regulation in human plasma (levels are typically maintained between 3.5 and 5.0 mmol L^−1^) in order to maintain a range of vital physiological processes, such as systemic blood-pressure control, glucose and insulin metabolism, renal concentrating ability, fluid and electrolyte balance, etc. (Gumz et al. 2015).

The estimated internal radiation dose due to ^137^Cs in 100 g portions of blanched, blanched and pickled and macerated mushrooms were low and in the range of 0.001 to 0.010 μSv (0.052 to 0.52 μSv weekly on an annual basis) (Table 4). Thus, the effective yearly gamma exposure dose from the ingested radiocaesium present in 100 g or 52.18 × 100 g portions of blanched, blanched and pickled and macerated mushrooms were considerably below the guidance dose of 1 mSv per year from ingestion by a representative individual (IAEA 2016).

Potassium concentrations in processed *B*. *edulis* varied from 2,200 to 3,700 mg kg^−1^ ww, and from 1,800 to 4,000 mg kg^−1^ ww in *Leccinum* spp. (Table 3). A 100 g portion (ww) of the studied blanched or pickled mushrooms could provide from 180 to 400 mg of K, with the median value of 330 mg accounting for 7% of the adequate daily intake (recommended intake = 4,700 mg) for adults, assuming that the absorption rate was around 90% (NIH 2019).

## Conclusions

Reductions (based on dw) of ^137^Cs and ^40^K (total K), respectively, for each of the different processing techniques studied, ranged from 23 to 43% and 24 to 51% after maceration, from ~ 3.8 to ~ 48% and 33 up to 41% after blanching and from 41 to 65% and 62 to 74% after blanching and pickling. It should be noted that any losses during maceration of dried mushrooms can be illusory if the water phase is preserved and consumed (in traditional recipes macerates are not discarded). The results of this study show that blanching of fresh mushrooms using traditional methods during household culinary processing may not be as efficient at removing the radioactivity resulting from ^137^Cs as has been shown in some other studies. When dried mushrooms are rehydrated, the initial rate of cell disintegration and other pre-preparation procedures used can affect the rate at which water-soluble metallic elements are leached out. Domestic procedures that are traditionally used for preparing mushrooms, such as blanching, pickling and macerating result in the loss of caesium and potassium at roughly the same rate. Mushrooms that are uncontaminated or contaminated with low levels of radiocaesium can still be high in dietary potassium when subjected to blanching and pickling.

## Data Availability

Not applicable.

## Appendix

**Table 5:** ^40^K and ^137^Cs activity concentration in fresh (lyophilised) and culinary processed caps, stipes and whole fruiting bodies of bolete mushrooms (Bq kg^-1^ on dry weight basis)

Species and kind of a culinary process	^40^K (Bq kg^-1^ dw)				^40^K; effect (decrease in %)			^137^Cs (Bq kg^-1^ dw)				^137^Cs; effect (decrease in %)		
	Caps	Stipes	Whole mushrooms	Q_C/S_	Caps	Stipes	Whole mushrooms	Caps	Stipes	Whole mushrooms	Q_C/S_	Caps	Stipes	Whole mushrooms
*Boletus edulis*														
Fresh → lyophilised ^*^	1,300 ± 120	1,300 ±170	1,300 ± 140	1.0	NA	NA	NA	110 ± 4	83 ± 4	90 ± 4	1.3	NA	NA	NA
Fresh → blanched	930 ± 110	520 ± 260	770 ± 180	1.8	29	60	41	61 ± 3	51 ± 4	57 ± 4	1.2	45	39	37
Fresh → blanched → pickled	610 ± 450	360 ± 130	510 ± 280	1.7	53	72	61	35 ± 7	28 ± 2	33 ± 4	1.2	68	66	63
Fresh → lyophilised (ground) → macerated	660 ± 100	590 ± 210	630 ± 150	1.1	49	55	51	69 ± 3	25 ± 3	51 ± 3	2.8	37	70	43
*Leccinum scabrum*														
Fresh → lyophilised ^*^	1,200 ± 110	1,000 ± 130	1,100 ± 120	1.2	NA	NA	NA	26 ± 2	10 ± 2	20 ± 2	2.6	NA	NA	NA
Fresh → blanched	650 ± 120	700 ± 180	670 ± 140	0.93	46	30	39	18 ± 2	< 13	~ 13	WD	31	WD	~ 31
Fresh → blanched → pickled	630 ± 170	670 ± 350	650 ± 240	0.94	48	33	41	9.9 ± 2.1	4.0 ± 3.0	7.5 ± 2.5	2.5	62	60	62
Fresh → lyophilised (ground) → macerated	1,100 ± 250	440 ± 130	840 ± 200	2.5	8	56	24	20 ± 4	7.7 ± 1.8	15 ± 3	2.6	23	23	23
*Leccinum versipelle*														
Fresh → lyophilised ^*^	1,200 ± 120	1,100 ± 140	1,200 ± 130	1.1	NA	NA	NA	17 ± 2	10 ± 2	14 ± 2	1.7	NA	NA	NA
Fresh → blanched	740 ± 210	900 ± 140	800 ± 180	0.82	38	18	33	8.8 ± 2.0	< 8.2	~ 7	WD	48	WD	~ 48
Fresh → blanched → pickled	330 ± 190	420 ± 130	370 ± 160	0.79	73	62	65	11 ± 3	3.9 ± 3.0	3.7 ± 3.0	2.8	35	67	74
Fresh → lyophilised (ground) → macerated	1000 ± 100	620 ± 120	850 ± 110	1.6	17	44	29	9.8 ± 1.8	8.8 ± 1.8	9.4 ± 1.8	1.1	42	12	32

Notes: ^*^Reference material; NA (not applicable); WD (without data).

**Table 6:** Potassium concentration in fresh and culinary processed caps, stipes and whole fruiting bodies of bolete mushrooms (mg kg^-1^ on dry weight basis)

Species and kind of a culinary process	K		
	Caps	Stipes	Whole mushrooms
*Boletus edulis*			
Fresh → lyophilised	46,000	46,000	46,000
Fresh → blanched	33,000	19,000	27,000
Fresh → blanched → pickled	22,000	13,000	18,000
Fresh → lyophilised (ground) → macerated	24,000	21,000	22,000
*Leccinum scabrum*			
Fresh → lyophilised	43,000	36,000	39,000
Fresh → blanched	23,000	25,000	24,000
Fresh → blanched → pickled	22,000	24,000	23,000
Fresh → lyophilised (ground) → macerated	39,000	16,000	30,000
*Leccinum versipelle*			
Fresh → lyophilised	43,000	38,000	43,000
Fresh → blanched	26,000	32,000	29,000
Fresh → blanched → pickled	12,000	15,000	13,000
Fresh → lyophilised (ground) → macerated	36,000	22,000	30,000

**Table 7:** Integrated data on effect of culinary processing on activity concentration ^40^K and ^137^Cs of *B. edulis*, *L. scabrum* and *L. versipelle* (percentage of decrease; mean ± S.D.; on dry weight basis)

Kind of a culinary process	Effect (decrease in %)					
	^40^K			^137^Cs		
	Caps	Stems	Whole mushrooms	Caps	Stems	Whole mushrooms
Fresh → blanched	38 ± 8	36 ± 22	38 ± 4	41 ± 9	39	39 ± 9
Fresh → blanched → pickled	58 ± 13	56 ± 20	56 ± 13	55 ± 18	64 ± 4	66 ± 7
Fresh → lyophilised (ground) → macerated	25 ± 22	52 ± 7	35 ± 14	34 ± 10	35 ± 31	33 ± 10
